# *Hermetia illucens* and *Tenebrio molitor* as Supplements in Maize Silage for Potential Use in Waterfowl Diets: Effects on Nutritional Composition, Selected Microbial Indicators, and Selected Mycotoxins

**DOI:** 10.3390/ani16091418

**Published:** 2026-05-06

**Authors:** Matúš Džima, Miroslava Kačániová, Daniel Bíro, Milan Šimko, Branislav Gálik, Michal Rolinec, Ondrej Hanušovský, Miroslav Juráček

**Affiliations:** 1 Department of Animal Nutrition, Institute of Nutrition and Genomics, Faculty of Agrobiology and Food Resources, Slovak University of Agriculture in Nitra, Tr. A. Hlinku 2, 94976 Nitra, Slovakia; xdzima@uniag.sk (M.D.); daniel.biro@uniag.sk (D.B.); branislav.galik@uniag.sk (B.G.); michal.rolinec@uniag.sk (M.R.); ondrej.hanusovsky@uniag.sk (O.H.); 2 Institute of Horticulture, Faculty of Horticulture and Landscape Engineering, Slovak University of Agriculture in Nitra, Tr. A. Hlinku 2, 94976 Nitra, Slovakia; miroslava.kacaniova@uniag.sk; 3 School of Medical & Health Sciences, VIZJA University, Okopowa 59, 01043 Warszawa, Poland

**Keywords:** corn silage, silage additives, yellow mealworm, black soldier fly, lauric acid, urea, microbiology, poultry nutrition

## Abstract

Alternative protein sources are increasingly considered in poultry nutrition, and insect meals may represent a promising strategy for modifying the compositional profile of maize silage in the context of waterfowl feeding. This study evaluated maize silage supplemented with *Hermetia illucens* (BAHI) or *Tenebrio molitor* (BATM) meal as alternatives to urea (BAUR) as a nitrogen source, including variants with lauric acid (BALA) and with the lactic acid bacteria (LAB) inoculant alone (BA), focusing on nutritional composition, microbiological quality, and selected mycotoxins (MT). Maize forage treated with LAB was ensiled for 8 weeks in five variants, with three independent silage replicates per treatment, and the silages were analyzed for nutrients, energy value, amino acids, fatty acids, microbial and MT content. Insect meals increased crude protein compared with BA (64.94–67.32 vs. 49.73 g/kg dry matter, DM) and nearly doubled ether extract in BAHI (40.08 vs. 21.74 g/kg DM), while apparent metabolizable energy for poultry ranged from 8.55 to 9.87 MJ/kg DM (BAHI). Urea elevated crude protein content to 66.37 g/kg DM without improving the essential amino acid index. Microbial indicators did not differ among treatments. In the MT profile, lower deoxynivalenol concentrations were observed in BAUR, BAHI, and BATM, and lower fumonisin B1 concentrations were observed in BATM and BALA, while aflatoxins and ochratoxin A were not detected. Overall, the results indicate that low-level inclusion of insect meals in combination with LAB improved the compositional characteristics of maize silage, while no significant differences were detected in the monitored microbial counts and only selective changes were observed in the mycotoxin profile.

## Simple Summary

Maize silage is a suitable forage source for geese and ducks, but its value in waterfowl feeding is limited by low protein and moderate energy concentration. In this study, maize forage was ensiled with a lactic acid bacteria inoculant alone and in combination with urea, *Hermetia illucens* meal, *Tenebrio molitor* meal, and lauric acid. The aim was to determine whether the addition of insect meals, urea, or lauric acid to a lactic acid bacteria-based silage treatment could improve the compositional profile of maize silage and influence its selected microbiological indicators or mycotoxin profile. Both insect meals in combination with LAB increased crude protein, and the *H. illucens* treatment also increased ether extract and calculated apparent metabolizable energy, N-corrected for poultry. Microbiological indicators were not significantly altered, while lower deoxynivalenol and fumonisin B1 concentrations were observed in some treatments. This study was designed as a silage characterization study and not as an in vivo feeding trial. These findings indicate that insect meal supplementation in combination with lactic acid bacteria improved selected compositional characteristics of maize silage under the present experimental conditions. However, its practical relevance for waterfowl feeding must be confirmed in future in vivo studies.

## 1. Introduction

Maize silage is an economical forage resource that may be useful in waterfowl feeding systems, especially where forage can partly reduce dependence on concentrated feeds. However, evidence in ducks and geese remains limited and not fully consistent. In geese, maize silage included at moderate levels did not impair growth performance and improved economic efficiency, whereas higher replacement levels reduced body weight gain [1,2]. In broiler ducks, maize silage combined with restricted commercial feeding improved feed conversion ratio and profitability without adverse effects on carcass traits [3]. These findings indicate that maize silage can be incorporated into waterfowl diets, but its value depends on inclusion level and overall diet formulation. From a practical perspective, maize silage may also improve feeding economics because it can partially replace concentrate feed when used at appropriate inclusion levels. In geese, moderate inclusion of whole-plant maize silage improved economic return, with the best result reported at 30% inclusion, while in broiler ducks feeding based on silage reduced commercial feed use and increased estimated profit relative to a standard commercial diet [2,3]. Silage may also be advantageous as a forage source, particularly during periods when fresh forage is limited, such as winter feeding conditions in geese [1,2]. However, because maize silage is primarily a forage component with limited protein density, its combination with protein-rich additives is nutritionally justified. In poultry nutrition, insect biomass is valuable not only as a source of crude protein, but also as a source of digestible essential amino acids, which is highly relevant because modern poultry diets are formulated on a digestible amino acid basis rather than on crude protein alone [4]. Insect meals generally meet poultry requirements in terms of amino acid composition and nutrient digestibility and can serve as partial substitutes for conventional protein sources in poultry diets [5]. Therefore, the inclusion of insect biomass in maize silage may represent a strategy for improving the compositional protein profile of a relatively protein-poor forage and make it nutritionally more relevant for poultry feeding systems [2,5].

Conventional protein sources used in poultry feeding remain under economic and sustainability pressure, which has increased interest in alternative feed materials such as insects [6,7]. Among the insect species considered most relevant for feed use are black soldier fly larvae (*Hermetia illucens*) and yellow mealworms (*Tenebrio molitor*) [6,7,8]. *H. illucens* is characterized by a high protein content and a lipid fraction rich in lauric acid, whereas *T. molitor* is also recognized as a protein rich insect material suitable for feed applications [9,10]. Because these species differ in nutrient composition and lipid profile, they may also differ in their nutritional and hygienic effects when incorporated into feeds.

The potential of insect material in silage is supported by ruminant studies showing that inclusion of *H. illucens* larvae increased crude protein (CP) content in total mixed ration silages [11]. Fermentation is also relevant in the insect value chain, where it may improve substrate utilization and product quality in some applications [12]. In *H. illucens*, the high proportion of lauric acid raises the possibility that this species may affect not only nutritional value, but also the microbiological and hygienic profile of maize silage [10].

*H. illucens* and *T. molitor* have been evaluated mainly in broiler chickens as partial replacements for conventional protein sources, and the available evidence supports their use primarily at low to moderate dietary inclusion levels. In a 42-day semi-commercial trial, 2% and 4% full fat *H. illucens* or *T. molitor* meals did not impair body weight gain, feed intake, feed conversion ratio, or mortality, while breast yield was higher in all insect-fed groups than in the control [13]. With partially defatted *H. illucens* meal, 5% and 10% inclusion supported favourable growth responses, whereas 15% impaired feed conversion and intestinal morphometry, suggesting that lower inclusion levels are more suitable [14]. For *T. molitor*, 2.5% inclusion improved starter body weight gain and reduced starter feed conversion ratio without negative effects on intestinal morphology [15]. In another broiler trial, 5% and 10% whole *T. molitor* larvae improved body weight gain, and 10% also increased carcass yield [16]. However, responses were not uniform. Šťastník et al. [17] found no improvement in feed conversion or carcass yield and reported a higher final live weight in the control group. A very high inclusion of *H. illucens* appears less suitable, since replacement of more than 50% of soybean meal protein reduced final body weight and worsened carcass and meat quality traits [18]. Although most available evidence on insect meals in poultry comes from broiler chickens, some data are also available in waterfowl. In Muscovy ducks, partially defatted *H. illucens* larva meal up to 9% did not impair growth performance, digestibility, slaughter traits, or meat quality, although meat fatty acid profile was affected [19,20]. In Sichuan White Geese, 1% *H. illucens* larvae meal was associated with improved growth and selected immune and intestinal traits after avian influenza vaccination [21]. However, evidence in waterfowl remains limited and fragmented compared with broiler chickens.

Unlike dry feed systems, maize silage undergoes fermentation under acidic and anaerobic conditions, which may influence fungal activity and the occurrence of mycotoxins differently. In addition, lactic acid bacteria used as silage inoculants may also contribute to changes in mycotoxin occurrence [22,23,24]. Feed safety remains an important consideration when novel materials are incorporated into poultry diets, particularly in preserved feeds where microbiological quality and mycotoxin (MT) occurrence directly affect practical usability. Insects can metabolize several MT, including aflatoxins (AFs), zearalenone (ZEA), deoxynivalenol (DON), fumonisins (FUMs), and ochratoxins (OTAs), although the pathways and toxicological implications are not yet fully resolved [25]. In addition, *T. molitor* meal did not impair monitored feed quality parameters in layer feed mixtures during storage under the tested conditions [26]. Despite growing interest in insect-based feeds, information is still lacking on maize silage supplemented with *H. illucens* meal or *T. molitor* meal, especially when nutritional composition, microbiological quality, and MT occurrence are evaluated together.

From a practical perspective, the use of maize silage in waterfowl feeding systems may reduce the reliance on commercial concentrate feeds, which represents a major cost component in poultry production. However, the relatively low protein content of maize silage limits its nutritional value and requires additional protein supplementation. Incorporating insect-derived biomass directly into silage may represent a strategy to improve its protein value using locally available and potentially sustainable resources, while maintaining the economic advantages of silage-based feeding systems.

The aim of this study was to investigate whether the inclusion of *Hermetia illucens* and *Tenebrio molitor* meals, in combination with lactic acid bacteria, can improve selected compositional parameters of maize silage and influence its microbiological quality and mycotoxin profile.

## 2. Materials and Methods

### 2.1. Sample Preparation

Maize hybrid Kathedralis (*Zea mays* L., FAO 480, dent type) was used in the experiment. It is a late two-line stay-green silage maize hybrid, suitable for sugar beet and maize production areas. It was ensiled in cooperation with PD Kozárovce in the Levice District, western Slovakia (48°18′47.7″ N, 18°30′31.6″ E). It was used because it was grown on the cooperating farm and considered suitable for the local growing conditions. The crop was harvested at the milk to dough stage using a self-propelled forage harvester (KRONE BIG X, Krone, Spelle, Germany) set to a theoretical chop length of 15 mm. DM content at harvest was 34.09%. A representative sample of fresh maize forage was collected before ensiling for chemical analysis. A biological silage additive was applied during harvesting by an applicator mounted on the forage harvester. The treated forage was then transported to the Laboratory of Feed Conservation, Department of Animal Nutrition, Institute of Nutrition and Genomics, Faculty of Agrobiology and Food Resources, Slovak University of Agriculture, Nitra, where the ensiling experiment was carried out. All variants were prepared from the same batch of harvested forage.

Five silage variants were prepared, each of three independent replicates. The treatments were maize forage with biological additive alone (BA), maize forage with biological additive and urea (BAUR), maize forage with biological additive and full fat *H. illucens* meal (BAHI), maize forage with biological additive and full fat *T. molitor* meal (BATM), and maize forage with biological additive and lauric acid (BALA). The urea (BAUR) treatment was included for comparison with insect meals (BAHI, BATM) as a CP source, whereas the lauric acid treatment (BALA) was included for comparison with *H. illucens* due to its high lauric acid content. Under EU legislation, urea is authorized only for ruminants with a functional rumen and therefore not for poultry, whereas lauric acid is authorized as a sensory additive in the functional group of flavouring compounds for all animal species [27,28,29].

The biological additive contained *Lentilactobacillus buchneri*, *Lacticaseibacillus rhamnosus*, and *Lactiplantibacillus plantarum* at a minimum concentration of 3.0 × 10^11^ LAB/g and was applied at 1 g/t fresh matter (FM), corresponding to a minimum delivered dose of 3.0 × 10^8^ CFU/kg FM. Urea was added at 0.3%, corresponding to 3 g/kg FM and 1.35 g N/kg FM. Nitrogen equivalence was used to standardize nitrogen input among treatments. Urea served as a conventional reference nitrogen additive in maize silage, whereas insect meals were included as alternative nitrogen sources for comparison. This design enabled comparison at a similar nitrogen input level, without implying equivalence in amino acid quality or energy value. Based on the analyzed CP content (Table 1) of the insect meals, nitrogen concentrations were calculated as 53.63 g/kg for *H. illucens* meal and 69.79 g/kg for *T. molitor* meal. On a nitrogen-equivalent basis in comparison with urea, insect meal inclusion rates were set at 25.17 g/kg FM for *H. illucens* and 19.34 g/kg FM for *T. molitor*. Lauric acid in the BALA treatment was added at a level equivalent to that supplied by *H. illucens* meal. The calculation was based on the analyzed ether extract (EE) and lauric acid content (Table 1) of *H. illucens* meal. Total fatty acids (FAs) in the lipid fraction were estimated using a conversion factor of 0.95 [30], and the lauric acid equivalent was calculated as 1.78 g/kg fresh matter. Whole full fat larvae were ground through a 1 mm sieve before use. The insect meals and maize matter were analyzed before ensiling to determine DM, CP, EE and lauric acid content.

All additives were mixed manually with the maize forage until visually homogeneous. Each treatment was ensiled in 3.5 L plastic silage units packed in vacuum bags. The bags were sealed with a vacuum sealer (MSW Motor Technics, Berlin, Germany) and stored at 21 °C for 8 weeks. After storage, the silage units were opened and representative samples were collected. Samples intended for chemical analysis were pre-dried at 55 ± 5 °C and ground to pass a 1 mm sieve (Fritsch, Idar-Oberstein, Germany).

### 2.2. Chemical Analysis

For maize forage, *H. illucens* meal, *T. molitor* meal, and the three independent samples of silage treatments were collected for chemical and microbiological analyses. MT analysis followed a different sampling design and is described separately below. Chemical composition was determined in the Laboratory of Feed Quality and Nutritional Value, Institute of Nutrition and Genomics, Faculty of Agrobiology and Food Resources, Slovak University of Agriculture, Nitra, using standard AOAC procedures [31].

Dry matter (DM) was determined gravimetrically at 103 ± 2 °C. CP was measured by the Kjeldahl method using a conversion factor of N × 6.25 (Kjeltec, FOSS Tecator, Hillerød, Denmark). EE was determined by Soxhlet extraction (Soxtec, Tecator, Denmark). Ash was determined by combustion in a muffle furnace at 530 ± 20 °C (Nabertherm, Lilienthal, Germany). Starch was analyzed polarimetrically using an Automatic Digital Polarimeter P3002RS (Germany). Organic matter (OM), non-structural carbohydrates (NSCs), and nitrogen free extract (NFE) were calculated by difference according to the following equations:

NSC = DM − (CP + EE + NDF + Ash),(1)

NFE = DM − (CP + EE + CF + Ash),(2)

OM = DM − Ash.(3)

Fibre fractions were analyzed using an ANKOM 200 Fibre Analyzer (ANKOM Technology, Macedon, NY, USA) following procedures derived from Van Soest et al. [32]. Neutral detergent fibre (NDF) was determined using sodium lauryl sulphate, acid detergent fibre (ADF) using cetyltrimethylammonium bromide, and acid detergent lignin (ADL) as the residue remaining after hydrolysis of ADF in 72% sulphuric acid. Crude fibre (CF) was determined gravimetrically as the residue remaining after sequential hydrolysis with sulphuric acid and potassium hydroxide. Cellulose (CEL) and hemicellulose (HEM) were calculated as follows:

CEL = ADF − ADL,(4)

HEM = NDF − ADF.(5)

FA composition was determined by gas chromatography (Agilent 7890A/6890A GC, Santa Clara, CA, USA) and expressed as relative proportions of individual FAs, including capric acid, lauric acid, myristic acid, palmitic acid, palmitoleic acid, stearic acid, oleic acid, linoleic acid, α-linoleic acid, arachidic acid, cis-11-eicosenoic acid, behenic acid, unidentified FAs, as well as grouped FA classes: saturated fatty acids (SFAs), monounsaturated fatty acids (MUFAs), polyunsaturated fatty acids (PUFAs), unsaturated fatty acids (UFAs), n3/n6 and n6/n3.

The amino acid (AA) profile was determined by ion-exchange chromatography using an Amino Acid Analyzer AAA 400 (INGOS, Prague, Czech Republic). The following AAs were quantified: alanine (Ala), aspartic acid (Asp), cysteine (Cys), glutamic acid (Glu), glycine (Gly), proline (Pro), serine (Ser), tyrosine (Tyr), arginine (Arg), histidine (His), isoleucine (Ile), leucine (Leu), lysine (Lys), methionine (Met), phenylalanine (Phe), threonine (Thr), and valine (Val). Total essential amino acids without tryptophan (SUMEAAs without Trp) and total nonessential amino (SUMNEAAs) acids were calculated as:

SUMEAAs (without Trp) = Arg + His + Ile + Leu + Lys + Met + Phe + Thr + Val,(6)

SUMNEAAs = Ala + Asp + Cys + Glu + Gly + Pro + Ser + Tyr.(7)

The protein quality of the tested silages was assessed by the essential amino acid index (EAAI_egg). This index was calculated as the geometric mean of the ratios of individual EAAs in the evaluated protein to those in the reference protein, following the method described by Oser [33], as applied in recent studies [34]:

(8) $$\mathrm{EAAI}\;=\;\sqrt[{n}]{\left(\frac{a_{1}}{r_{1}}\right)\;\times \;\left(\frac{a_{2}}{r_{2}}\right)\;\times \ldots \times }\;\left(\frac{a_{n}}{r_{n}}\right).$$

EAAI—essential amino acid index, a_i_—concentration of the *i*-th essential amino acid in the evaluated protein, r_i_—concentration of the *i*-th essential amino acid in the reference egg protein, and n—number of essential amino acids included in the index.

The AA profile of duck and goose egg protein reported by Vojtaššáková et al. [35] was used as the reference. Subsequently, EAAI_D_, the essential amino acid index calculated against duck egg protein, and EAAI_G_, the essential amino acid index calculated against goose egg protein, were calculated. The EAAI was calculated against these reference proteins because the evaluated silages were considered in the context of potential use in waterfowl feeding, and duck and goose egg protein therefore represented biologically relevant reference standards.

The apparent metabolizable energy corrected for nitrogen (AMEN) was calculated according to Mariano et al. [36] using a regression equation based on EE, CF, NDF and ash content, expressed as % of DM:

AMEN = 4 164.187 + 51.006 × % EE − 197.663 × % Ash − 35.689 × % CF − 20.593 × % NDF.(9)

The calculated values were expressed as kcal/kg DM and converted to MJ/kg using a conversion factor of 0.004184.

**Table 1:** Basic nutritional parameters of evaluated feed materials.

	DM	CP	EE	C12:0
	g/kg	g/kg DM		%
Maize forage	340.88 ±1.49	47.73 ±0.56	18.33 ±0.14	N.D.
*H. illucens*	960.25 * ±0.96	349.05 ±0.95	340.12 ±2.32	22.76 ±0.35
*T. molitor*	952.55 * ±1.12	457.92 ±1.79	311.01 ±7.97	0.34 ±0.01

Abbreviations: DM—dry matter, CP—crude protein, EE—ether extract, C12:0—lauric acid, N.D.—not detected. * DM values for *H. illucens* and *T. molitor* were previously published in Džima et al. [37].

### 2.3. Mycotoxin Analysis

For MT analysis, three samples of insect meals and fresh maize forage were pooled into one composite sample for each material and analyzed once. In contrast, silage mycotoxin concentrations were determined in three independent samples per treatment. The content of selected MTs relevant to European union (EU) feed legislation was determined in cooperation with the accredited laboratory Romer Labs Diagnostic GmbH (Tulln, Austria). The analyzed MTs included fumonisin B1 (FB1), fumonisin B2 (FB2), total aflatoxins (ΣAFs), ochratoxin A (OTA), zearalenone (ZEA), T-2 toxin (T-2), HT-2 toxin (HT-2), and deoxynivalenol (DON). Total aflatoxins were calculated as:

ΣAFs = AFB1 + AFB2 + AFG1 + AFG2.(10)

Analysis was performed by HPLC MS/MS using the Romer Labs Diagnostic GmbH, (Tulln, Austria) procedure AT SOP 31 based on EN 17280:2019 [38]. Samples of 10 g were extracted with 30 mL acetonitrile/water (7:3, *v*/*v*), centrifuged, and diluted tenfold with HPLC eluent A. Internal standards were added before dilution. Chromatographic separation was performed on a Phenomenex Gemini C18 column (4.6 × 150 mm, 5 µm) using an Agilent 1260 Infinity II HPLC system coupled with HPLC MS/MS detection. Quantification was based on internal standard calibration using calibration curves constructed from analyte to internal standard signal ratios. Identification followed SANTE 12089/2016 based on retention time and two product ions. Two internal control samples were included in each analytical batch. Limits of detection were 0.3 µg/kg for AFB1, 0.5 µg/kg for AFB2, G1 and G2, 15 µg/kg for DON and HT-2, 10 µg/kg for FB1, FB2 and T-2, and 3 µg/kg for ZEA. MT concentrations were first determined on a laboratory dry matter basis and then recalculated to 88% DM, expressed as µg/kg.

### 2.4. Microbiological Analyses

Microbiological analyses focused on the total viable count (TVMC), lactic acid bacteria (LAB), coliform bacteria (CB), and microscopic filamentous fungi (MFF). For preparation of the primary dilution, 5 g of silage sample was homogenized with 45 mL of sterile 0.87% saline solution. Serial decimal dilutions (10^−2^ to 10^−4^) were subsequently prepared. The total viable count was determined by plating 100 µL aliquots onto Tryptic Soy Agar (TSA, Sigma-Aldrich^®^, St. Louis, MO, USA), followed by incubation at 30 °C for 48–72 h. CB were enumerated on MacConkey agar (MC, Sigma-Aldrich^®^, St. Louis, MO, USA) after incubation at 37 °C for 24–48 h. LAB were cultivated on MRS agar (Sigma-Aldrich^®^, St. Louis, MO, USA). Inoculated plates were incubated under microaerophilic conditions (5% CO_2_) at 37 °C for 72 h. MFF were determined on malt extract agar (MEA, Sigma-Aldrich^®^, St. Louis, MO, USA) supplemented with bromocresol green (0.020 g/L). Plates were incubated aerobically at 25 °C for 5 days. Microbial counts were expressed as colony forming units and subsequently converted to log CFU/g.

### 2.5. Statistical Analysis

Statistical analysis was performed using IBM SPSS Statistics 26.0 (IBM Corp., Armonk, NY, USA). Differences among treatments were evaluated by one-way analysis of variance followed by Tukey’s post hoc test. Results are presented as mean ± standard deviation (SD), and differences were considered significant at *p* < 0.05. Pearson correlation coefficients were calculated to assess relationships among selected variables. Correlation analysis was based on pairwise available observations, and only biologically relevant significant correlations were selected for presentation in the manuscript. Pearson correlation coefficients are presented as r. A heat map with hierarchical clustering was generated in SRplot software, ver. 2023 (NewCore Biotech, Shanghai, China; accessed on 10 March 2026), as a visual aid to support the interpretation of relationships among the analyzed variables [39].

## 3. Results

### 3.1. Basic Nutritional Composition and Energy Value

DM content differed significantly among treatments (Table 2). Both LAB + insect meal treatments showed higher DM than BA and BAUR, whereas the BALA had the lowest DM (*p* < 0.05). The overall variation was relatively small. Compared with BA, CP was higher in BAUR, BAHI, and BATM (*p* < 0.05), reflecting the effect of nitrogen supplementation. EE was lower in BAUR and higher in both insect meal treatments compared with BA (*p* < 0.05), whereas BALA did not differ from the BA. Starch content was reduced in all supplemented treatments compared with BA (*p* < 0.05), whereas NSC decreased only in the BATM and BALA treatments (*p* < 0.05). The BAHI treatment increased ash and decreased OM compared with the BA (*p* < 0.05), showing that its effect extended beyond protein and lipid enrichment. The BATM treatment had lower ash and higher OM than the BA (*p* < 0.05). Only the BAHI treatment increased calculated AMEN compared to BA (*p* < 0.05), whereas the BALA reduced it (*p* < 0.05).

### 3.2. Structural Carbohydrates

Structural carbohydrate fractions differed among treatments (Table 3). The *H. illucens* treatment reduced CF, NDF, ADL, and HEM (*p* < 0.05), resulting in a lower fibre profile than BA. The BATM treatment increased NDF (*p* < 0.05) but did not affect CF, ADF, ADL, and CEL. The BALA treatment showed the highest (*p* < 0.05) values of CF, NDF, ADF, and CEL among the tested variants. BAUR had lower (*p* < 0.05) ADL and higher (*p* < 0.05) CEL compared with BA, whereas the remaining fibre fractions did not differ (*p* > 0.05) compared with the control.

**Figure 1:**
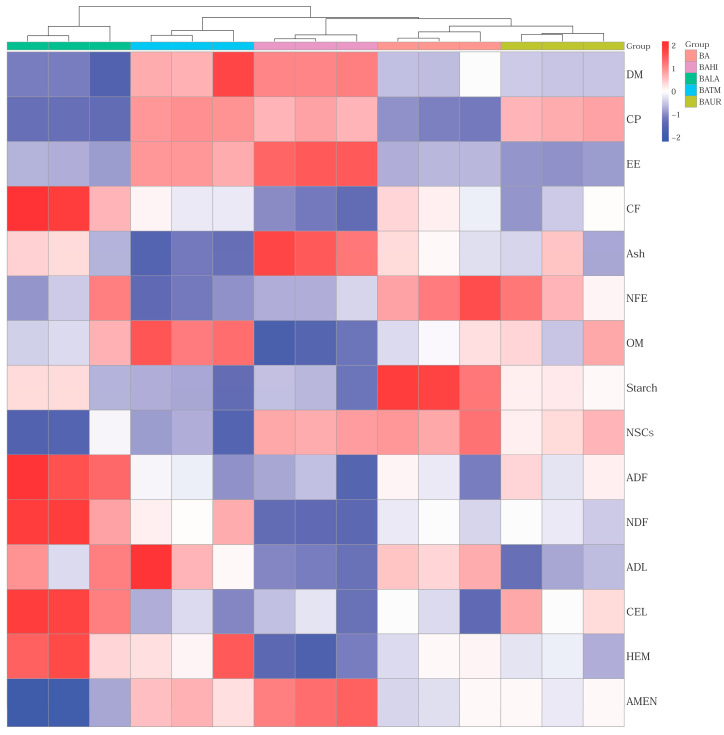
Heat map and clustering of compositional parameters of evaluated silage samples. Abbreviations: BA—biological additive, BAUR—BA + urea, BAHI—BA + *H. illucens*, BATM—BA + *T. molitor* meal, BALA—BA + lauric acid, DM—dry matter, CP—crude protein, EE—ether extract, CF—crude fibre, NFE—nitrogen free extract, OM—organic matter, NSCs—non-structural carbohydrates, ADF—acid detergent fibre, NDF—neutral detergent fibre, ADL—acid detergent lignin, CEL—cellulose, HEM—hemicellulose, AMEN—nitrogen-corrected apparent metabolizable energy for poultry.

The heat map (Figure 1) shows clear differences among treatments in nutrient profile composition, with the largest changes observed in the insect-containing variants, especially BAHI and BATM, as well as in BALA. BAHI and BATM were associated mainly with higher relative levels of DM, CP, EE, and AMEN, whereas BALA displayed a distinct profile that was more closely associated with fibre fractions, which indicates that both the extent and the direction of compositional changes varied among treatments. The heat map is presented primarily as a visual aid to support the interpretation of the compositional differences among treatments.

### 3.3. Amino Acid Composition and Protein Quality

Overall, the maize silages, regardless of the additive applied, were characterized by the highest content of Leu among the EAAs. Among the NEAAs, Glu was present at the highest concentration in all variants except BALA, in which Pro was the most abundant. Compared with BA, the sum of EAAs was higher in BAHI and BATM (Table 4; *p* < 0.05). The sum of NEAAs was also higher (*p <* 0.05) in BAHI, BATM, and BALA than in BA, whereas BAUR did not differ from BA (Table 5; *p* > 0.05). At the level of individual AAs, both insect meal treatments in combination with LAB affected a broad range of essential and non-essential AAs. Compared with BA, the LAB + *H. illucens* treatment increased (*p <* 0.05) all EAAs except Val, while Val decreased (*p* < 0.05). Among the NEAAs, it increased Ala, Asp, Glu, Gly, Ser, and Tyr (*p* < 0.05). The LAB + *T. molitor* treatment increased all EAAs and increased Ala, Asp, Glu, Gly, Pro, Ser, and Tyr among the NEAAs (*p* < 0.05). LAB + Urea did not affect the sum of essential or non-essential AAs compared with BA (*p* > 0.05), but both EAAIs were lower.

Protein quality indices showed the same treatment pattern. Compared with BA, both EAAI values decreased (*p <* 0.05) in BAUR, increased (*p <* 0.05) in BAHI and BATM, and did not differ in BALA (*p* > 0.05). The highest (*p <* 0.05) values were recorded in BATM.

**Figure 2:**
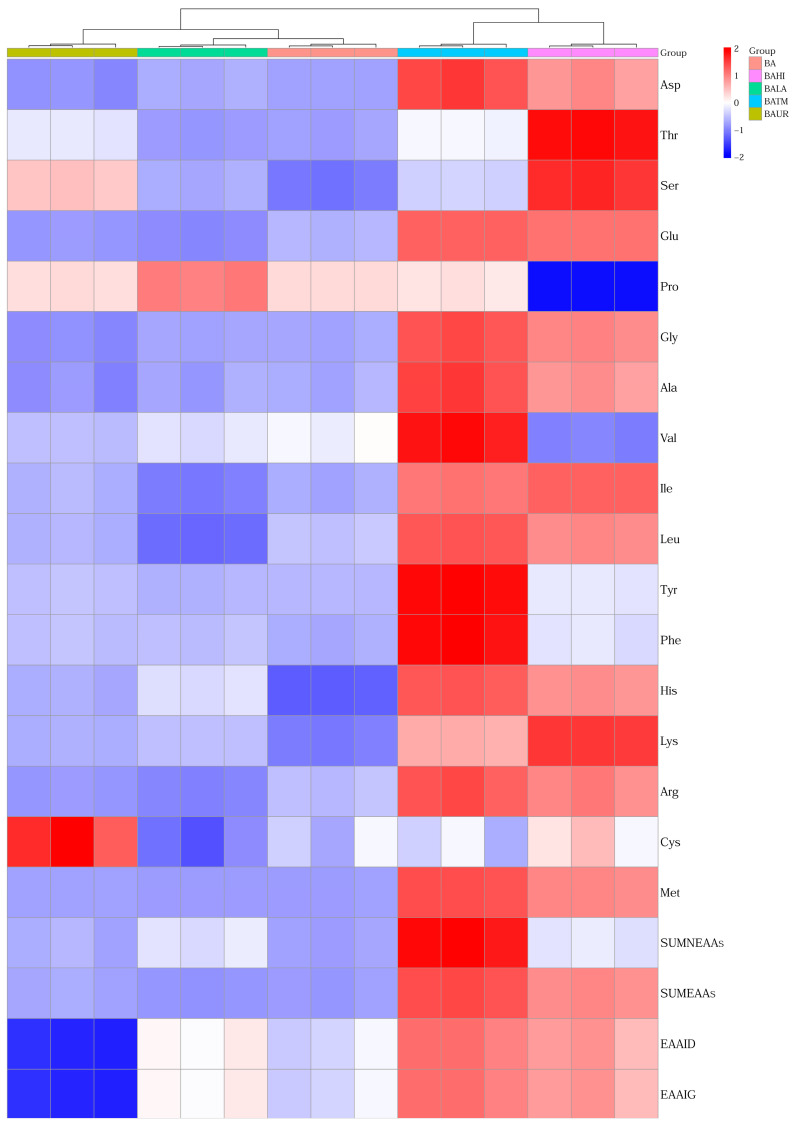
Heat map and clustering of amino acid parameters of evaluated silage samples. Abbreviations: BA—biological additive, BAUR—BA + urea, BAHI—BA + *H. illucens*, BATM—BA + *T. molitor* meal, BALA—BA + lauric acid, Asp—aspartic acid, Thr—threonine, Ser—serine, Glu—glutamic acid, Pro—proline, Gly—glycine, Ala—alanine, Val—valine, Ile—isoleucine, Leu—leucine, Tyr—tyrosine, Phe—phenylalanine, His—histidine, Lys—lysine, Arg—arginine, Cys—cysteine, Met—methionine, SUMNEAAs—total non-essential amino acids, SUMEAAs—total essential amino acids without tryptophan, EAAI_D_—essential amino acid index relative to duck egg protein, EAAI_G_—essential amino acid index relative to goose egg protein.

The heat map (Figure 2) showed a clear separation of treatments according to amino acid composition and protein quality indices, with the most distinct profiles observed in BATM and BAHI. BATM was associated with the highest relative levels of most essential and non-essential amino acids, as well as SUMEAAs, SUMNEAAs, EAAI_D_, and EAAI_G_, whereas BAHI showed a similar but generally less pronounced pattern.

### 3.4. Fatty Acid Profile

In all maize silage treatments, regardless of additive supplementation, linoleic acid was the most abundant fatty acid, followed by oleic acid, with palmitic acid ranking third. In the BAHI variant, an increase in lauric acid and total SFAs, together with a decrease in linoleic acid, α-linolenic acid, and total PUFAs was observed compared with BA (*p* < 0.05; Table 6). BAHI also had lower oleic acid than BA (*p* < 0.05). BATM had higher oleic acid and total MUFAs and lower linoleic acid and total PUFAs than BA (*p* < 0.05). Compared to BA, BATM did not differ in lauric acid. BALA increased (*p* < 0.05) lauric acid, total SFAs, α-linolenic acid, and the n3/n6 ratio and decreased (*p* < 0.05) oleic acid and linoleic acid in comparison with BA. BAUR differed from BA only in higher (*p* < 0.05) palmitic acid and SFAs.

**Figure 3:**
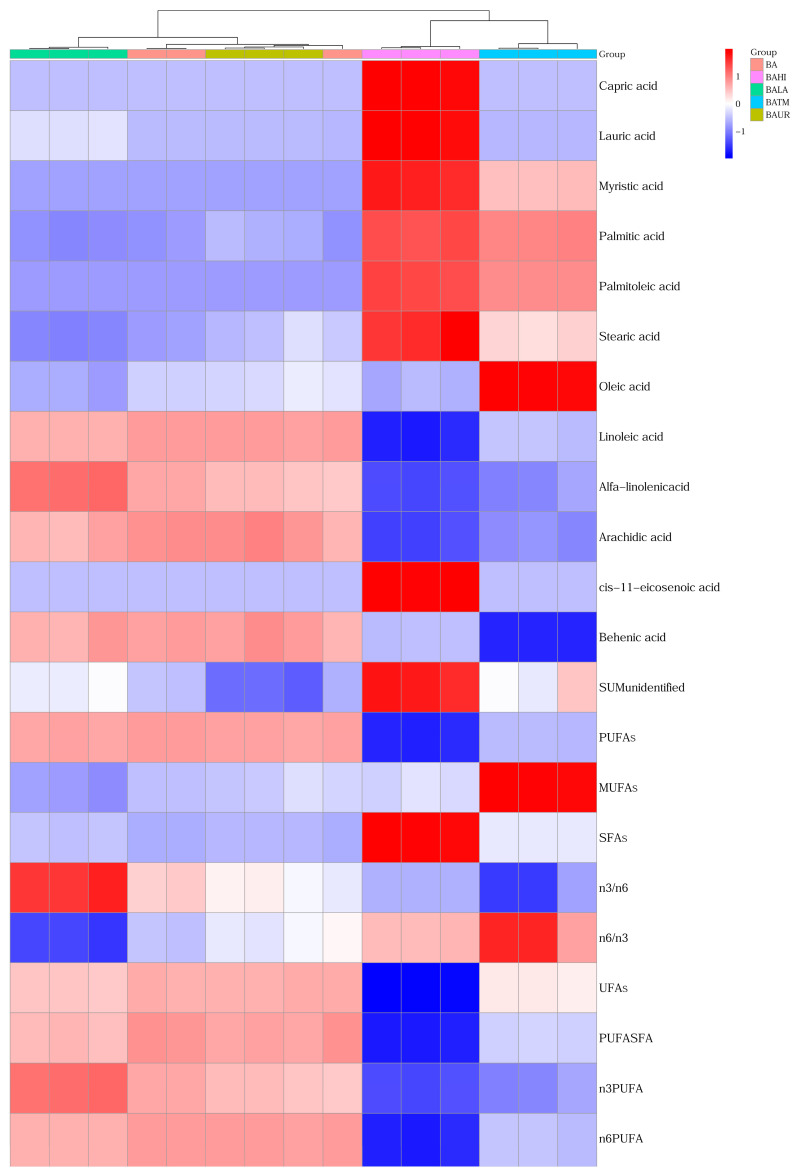
Heat map and clustering of fatty acids of evaluated silage samples. Abbreviations: BA—biological additive, BAUR—BA + urea, BAHI—BA + *H. illucens*, BATM—BA + *T. molitor* meal, BALA—BA + lauric acid, PUFAs—polyunsaturated fatty acids, MUFAs—monounsaturated fatty acids, SFAs—saturated fatty acids, UFAs—unsaturated fatty acids.

The heat map (Figure 3) showed clear treatment-related differences in the overall fatty acid profile, with the most distinct patterns observed in BAHI and BATM. These two variants were separated most clearly from the remaining treatments.

### 3.5. Selected Microbiological Indicators and Mycotoxins

No significant differences among treatments were observed in TVC, CB, LAB, and MFF (Table 7). None of the treatments significantly affected (*p* > 0.05) the culture-based microbiological indicators. The BAHI and BATM variants had the highest LAB counts among the treatments, although they did not differ significantly (*p* > 0.05) from all other treatments.

Treatment effects were more selective in the MT profile than in the microbiological indicators (Table 8). Of all the tested mycotoxins, only DON, FB1, and FB2 were detected in the corn silages overall, each with an incidence of 100%, whereas T-2 toxin was detected only in the BAHI variant, with an incidence of 33.33%. Regardless of the additive applied, the corn silages were most heavily contaminated with DON. Lower FB1 concentrations were observed in BATM and BALA than in BA (*p* < 0.05), whereas BAUR and BAHI did not differ from BA (*p* > 0.05). FB2 did not differ among treatments. Lower DON concentrations were observed in BAUR, BAHI, and BATM than in BA (*p* < 0.05), whereas BALA did not differ from BA (*p* > 0.05).

**Figure 4:**
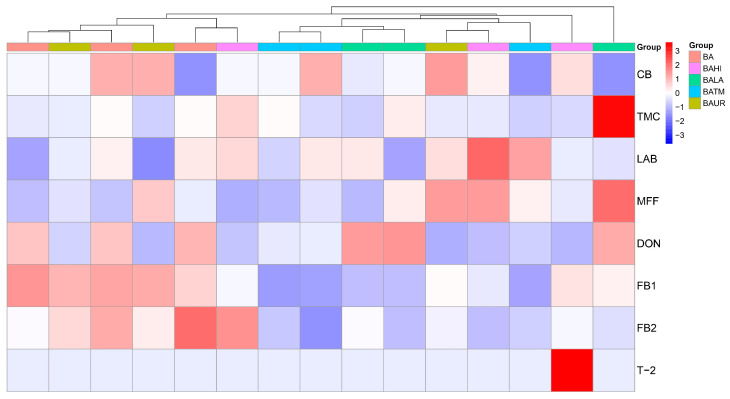
Heat map and clustering of microbial and mycotoxin parameters of evaluated silage samples. Abbreviations: BA—biological additive, BAUR—BA + urea, BAHI—BA + *H. illucens*, BATM—BA + *T. molitor* meal, BALA—BA + lauric acid, CB—coliform bacteria, TVC—total viable count, LAB—lactic acid bacteria, MFF—microscopic filamentous fungi, DON—deoxynivalenol, FB1—fumonisin B1, FB2—fumonisin B2, T-2—T2-toxin.

The heat map (Figure 4) revealed only partial separation of treatments, indicating that the overall microbiological and mycotoxin profiles were not completely distinct among variants. The greatest variation was associated mainly with DON, FB1, FB2, and T-2, whereas T-2 occurred as a clear isolated signal in only one sample, and FB1 and DON showed more pronounced treatment-related differences.

### 3.6. Selected Correlations Among Nutritional, Microbiological and Mycotoxin Variables

DON was strongly negatively correlated with CP and strongly positively correlated with fibre fractions (NDF, ADF, HEM), indicating that higher DON concentrations occurred in variants with higher fibre content. MFF were not significantly correlated with DON, FB1, or FB2, indicating that culture-based fungal counts were not related to the measured MT concentrations. CP was strongly positively correlated with SUMEAAs. ADF was strongly negatively correlated with EE. NDF showed moderate negative correlations with both EE and CP. A strong positive correlation was also observed between FB1 and FB2.

## 4. Discussion

### 4.1. Nutritional Enrichment of Maize Silage by Insect-Derived Additives

The literature indicates that the recommended DM content of maize intended for ensiling is approximately within the range of 300 to 350 g/kg [40,41]. The maize biomass used in this study had a DM content of 340.88 g/kg. The DM content in the tested maize silages ranged from 326.15 (BALA) to 350.28 (BATM) g/kg. The highest DM content was observed in BAHI and BATM, which may be linked to the high DM content of used additives (*H. illucens*, *T. molitor*). Yang et al. [42] also reported that in whole plant maize silage, the application of *L. plantarum* increased DM content from 352.4 to 365.1 g/kg, while the application of a combination of *L. lactis* + *L. buchneri* increased it to 373.6 g/kg. In maize silage, Jatkauskas et al. [43] found that the application of a combination of *L. buchneri* and *L. lactis* resulted in a 1.5% higher average dry matter content compared with the uninoculated control.

The CP content of maize silage may range approximately from 47 to 93.8 g/kg DM, depending on genotype and ensiling conditions [44,45,46]. The CP content in the evaluated silages ranged from 47.81 (BALA) to 67.32 (BATM) g/kg DM. Urea and both insect meals increased CP in tested silage samples. A comparable nutrient-enriching role of insect material has been reported in total mixed ration silages containing *H. illucens*, where CP increased from 14.06 to 15.87% DM before ensiling and from 12.08 to 15.26% DM after ensiling using defatted *H. illucens* treatment [11]. After the application of *L. buchneri* and *L. lactis*, the CP content in maize silage was on average 10.8% higher, probably as a result of lower proteolysis [43]. A similar trend was also reported by Chen et al. [47]. After inoculation with *L. plantarum*, *P. acidilactici*, *Enterococcus faecium*, or *Ligilactobacillus salivarius*, CP content increased from 83.95 to 85.92 g/kg DM, although this difference was not statistically significant.

EAAs are indispensable in poultry nutrition for growth, production, feed utilization, and body protein synthesis [4,48]. Lysine is important in duck nutrition for growth, muscle development, and feed utilization, and in laying ducks for egg production, hatchability, and the body weight of one-day-old ducklings [49,50]. Methionine is the first limiting AA in poultry, and in ducks significantly affects productive and reproductive performance, albumen formation, and antioxidant protection of the organism [51,52]. Threonine is important not only for growth but also for the intestinal barrier function and immunity, as it increases IgA concentration, and supports the development of immune organs in Pekin ducks [53,54]. Arginine is essential for poultry and in geese contributes to productive performance, antioxidant capacity, and immunity, while increasing IgA and IgG [55,56]. Luo et al. [57] reported that maize silage contained 1.8 g/kg DM arginine, 2.5 g/kg DM lysine, 1.1 g/kg DM methionine, and 2.9 g/kg DM threonine. In the experimental variants of maize silage, Lys content ranged from 1.62 (BA) to 3.09 (BAHI), Met from 1.41 (BA, BALA) to 3.76 (BATM), Thr from 1.23 (BALA) to 2.55 (BAHI), and Arg from 1.04 (BALA) to 1.74 (BATM) g/kg DM. The higher EAA content observed in the evaluated silages was associated with the inclusion of *H. illucens* and *T. molitor*, which are recognized as rich sources of amino acids compared with urea [58,59]. Corn silage treated with urea-based additives increased CP but was not accompanied by a parallel increase in AA content [60]. Also, Xu et al. [61] recorded a higher lysine concentration in maize silage after inoculation with *L. plantarum* and *L. buchneri* compared with the control, whereas Guo et al. [62] found the opposite trend for methionine after inoculation with *L. plantarum*. A microbial inoculum containing LAB did not significantly affect threonine or arginine content in maize silage [60].

The EAAI is a parameter used to evaluate the biological quality of proteins and takes into account the content of EAAs in relation to a reference protein [33]. However, maize silage is used primarily as an energy feed for ruminants with a relatively low CP content, while maize protein is deficient mainly in lysine and tryptophan. From a compositional perspective, supplementation of maize silage with larvae of *H. illucens* and *T. molitor* may improve its AA profile, as these insect products are recognized as valuable sources of protein and EAAs for poultry [5,63,64,65]. In this study, EAAI was calculated without Trp against duck and goose egg reference proteins because the evaluated silages were considered in the context of potential use in waterfowl feeding, and these proteins represented biologically relevant reference standards for this purpose. EAAI_D_ ranged from 48.31 (BAUR) to 72.86% (BATM). EAAI_G_ ranged from 46.15 (BAUR) to 69.60% (BATM), providing a comparative indication of AA balance among treatments in relation to these reference proteins.

NEAAs are not considered as nutritionally negligible in poultry nutrition because they are important for metabolic regulation, tissue development, and overall performance, and their importance may be even greater, particularly in low protein diets [4,66]. In poultry nutrition, glutamate is an important energy substrate for small intestinal enterocytes. Proline supports collagen and keratin synthesis, and alanine is involved in nitrogen transport and energy metabolism [4,66,67]. For maize silage, the literature reports values of 9.4 to 13.0, 5.1 to 6.3, and 6.1 to 7.1 g/kg DM for glutamic acid, proline, and alanine, respectively [68]. In experimental treatments, glutamate content ranged from 6.25 (BALA) to 8.90 (BATM), proline from 3.80 (BAHI) to 7.42 (BALA), and alanine from 3.82 (BALA) to 5.38 (BATM) g/kg DM. In maize silage, Wagali et al. [60] recorded a decreasing trend in some non-essential amino acids after the application of additives, especially under LAB + urea treatment, whereas Cleale et al. [69] did not demonstrate differences in total NEAAs after inoculation with *Pediococcus acidilactici* and *Lactobacillus xylosus* compared with the control. These changes indicate an improved amino acid profile of the evaluated silages, although their practical significance for waterfowl feeding needs to be confirmed in vivo.

Literature data indicate that the EE content of maize silage may range approximately from 15.7 to 43.0 g/kg DM [70,71]. The EE content in the tested silages ranged from 19.77 (BAUR) to 40.08 (BAHI) g/kg DM. The highest EE content in BAHI is linked to the addition of *H. illucens*, which is rich in EE. Current data on the effect of LAB on EE content in maize silage are not entirely consistent. Dong et al. [72] confirmed an increase in EE content after the application of a silage inoculant containing *L. plantarum*, *L. buchneri*, and *L. casei*, whereas Yang et al. [42] did not confirm a significant effect on EE content after the application of *L. plantarum* or a combination of *L. lactis* + *L. buchneri*.

The main essential fatty acids in poultry nutrition are omega-6 and omega-3 PUFAs, especially linoleic acid and α-linolenic acid, because the poultry organism cannot synthesize them de novo and they must be supplied through the diet. These fatty acids are important for the structure of cell membranes and affect productive performance, immunity, and reproduction [73,74]. Published studies on maize silage report that the content of palmitic, linoleic, and α-linolenic acids ranged approximately from 13.7 to 29.5, 18.7 to 48.6, and 3.4 to 11.1% of total fatty acids, respectively [75,76]. In the monitored samples, linoleic acid content ranged from 33.00 (BAHI) to 49.35 (BA), palmitic acid from 11.95 (BALA) to 15.10 (BAHI), and α-linolenic acid from 4.16 (BAHI) to 7.44 (BALA) % of FAs. According to Alves et al. [77], a bacterial inoculant in maize silage did not have a significant effect on the main fatty acids, whereas ensiling itself led to a decrease in their relative proportions, but not in their concentrations.

In waterfowl, PUFAs, MUFAs, and the n6/n3 ratio have been discussed in relation to immunity and lipid metabolism [78,79,80]. In the present study, these variables differed among silages according to the applied additive. In maize silage without additives, PUFAs accounted for 53.20 to 55.45%, MUFAs for 22.10 to 23.45%, and the n6/n3 ratio reached 6.60:1 to 6.68:1 of total fatty acids [76,78,79,80]. In the experimental silages, PUFA content ranged from 37.15 (BAHI) to 55.87 (BAUR), MUFAs from 24.67 (BALA) to 32.58 (BATM) %, and the n6/n3 ratio from 6.49:1 to 8.40:1. The effect of LAB on the fatty acid profile of maize silage is not consistent, as Alves et al. [77] did not demonstrate an effect of the inoculant, whereas Jalč et al. [81] recorded a lower n6/n3 ratio, lower MUFA content, and higher PUFAs after inoculation.

According to available data, the starch content in maize silages ranges from 96.3 to 463.6 g/kg DM, with its concentration being most strongly influenced by crop maturity at harvest [63,82,83]. Maize starch represents the primary energy source in poultry nutrition, and its digestive kinetics affect growth performance, postprandial glycemia, and nutrient utilization [84,85]. The starch content in the tested silages ranged from 240.98 (BATM) to 275.05 (BA) g/kg DM. The effect of LAB on starch content in maize silage was not consistent. Yang et al. [42] did not find significant changes in starch content after the application of *L. plantarum* or a combination of *L. lactis* + *L. buchneri*, whereas Xue et al. [86] recorded lower starch content after 90 days of ensiling in treatments with *L. plantarum* and *L. buchneri* than in the control.

The OM content in maize silages ranges approximately from 900 to 978 g/kg DM [83,87,88]. The OM content in the experimental silage treatments ranged from 959.03 (BAHI) to 962.65 (BATM) g/kg DM. The effect of LAB on OM content in maize silage was not consistent, as Xue et al. [86] did not observe a significant change in ash content, whereas Müller et al. [89] reported lower ash content after LAB application and thus, indirectly, a slightly higher OM content.

Energy represents one of the basic nutritional factors in duck and geese nutrition, as the level of metabolizable energy affects production parameters, feed intake, and the efficiency of utilization of other nutrients [90,91]. Khan et al. [92] reported that the metabolizable energy of maize silage ranged from 9.62 to 10.50 MJ/kg DM depending on genotype and harvest stage. In the monitored maize silages, calculated AMEN content ranged from 8.55 to 9.87 MJ/kg DM. The calculated AMEN content of the tested silages was increased by the addition of *H. illucens*, which may be related to the higher EE content in the insect meal. The effect of LAB on the metabolizable energy of maize silage was not clear, as Müller et al. [89] recorded slightly higher ME after the application of a mixture of *L. plantarum*, *L. brevis*, and *L. kefiri*, whereas Filya and Sucu [93] did not observe a demonstrable change after the application of several LAB inoculants. Thus, the observed differences indicate compositional changes in energy-related parameters rather than direct evidence of improved feeding performance.

### 4.2. Structural Carbohydrate Fractions

NDF represents a functionally important feed component in the nutrition of geese and ducks, as it affects growth, intestinal morphology, and the cecal microbiota, while in geese the potential utilization of hemicellulose as part of the fibre complex has also been suggested [94,95,96,97]. In maize silages, NDF content ranges from 294.9 to 687 g/kg DM, while hemicellulose content ranges from 261 to 325 g/kg DM [83,98]. In the experimental silages, NDF content ranged from 315.48 (BAHI) to 388.01 (BALA) g/kg DM, and hemicellulose content from 138.15 (BAHI) to 181.71 (BALA) g/kg DM. The effect of LAB on NDF and HEM content in maize silage was not consistent, as Müller et al. [87] recorded a decrease in NDF from 438 to 413 g/kg DM after the application of a mixture of *L. plantarum*, *L. brevis*, and *L. kefiri*, whereas Filya and Sucu [93] and Yang et al. [42] did not observe a significant effect of LAB on NDF, while Filya and Sucu [93] likewise did not find a significant change in hemicellulose content.

### 4.3. Microbiological Quality and Mycotoxin Profile

High counts of CB primarily indicate impaired hygiene and fecal contamination of the feed and, consequently, a higher risk of the presence of pathogenic enterobacteria, including pathogenic strains of *Escherichia coli*, which cause colibacillosis in poultry and is associated with mortality and reduced productivity. High counts of microscopic filamentous fungi reduce the nutritional quality of the feed and increase the risk of mycotoxicosis, leading to intestinal damage, impaired nutrient absorption, immunosuppression, and lower performance [99,100,101,102,103]. In the tested silages, TVC ranged from 1.42 (BAUR) to 1.89 (BALA), CB from 1.30 (BALA) to 1.41 (BAUR), and MFF from 1.40 (BA) to 1.62 (BAUR) log CFU/g. In the literature, the indicative reference values reported for the evaluation of the microbiological quality of feeds are approximately 3.0 × 10^6^ CFU/g for TVC, approximately 4 log CFU/g for CB, and approximately 2.0 × 10^5^ CFU/g for MFF [104,105]. When converted to logarithmic expression, these indicative literature limits correspond to values of approximately 6.48 log CFU/g for TVC, 4.00 log CFU/g for CB, and 5.30 log CFU/g for MFF. None of the selected monitored microbiological groups in the tested maize silage samples exceeded these indicative reference limits. The present microbiological evaluation was limited to culture-based counts of selected microbial groups and does not reflect the broader microbial community structure of the silages.

Among the available studies, the most directly comparable evidence for maize silage is provided by Kalúzová et al. [106], who reported that untreated corn silage contained 3.54 log CFU/g TVC, LAB ranging from 2.69 to 5.11 log CFU/g depending on the culture medium, undetectable CB, and 2.73 log CFU/g MFF. In silage treated with a LAB-based additive, TVC ranged from 3.23 to 3.51 log CFU/g, LAB from 2.83 to 5.05 log CFU/g, CB remained undetectable, and MFF ranged from 2.13 to 2.21 log CFU/g, whereas in urea-treated silage TVC reached 3.26 log CFU/g, LAB 4.14 to 4.21 log CFU/g, CB remained undetectable, and MFF increased to 3.38 log CFU/g. Additional maize silage studies showed that LAB inoculation may increase LAB counts and reduce yeast or filamentous fungi under specific conditions, but the response is not uniform across studies [107,108].

Only Fusarium mycotoxins were detected in the analyzed maize silages, with DON occurring at the highest concentration, followed by FB1 and FB2, both with 100% incidence, while T-2 toxin was detected in only one BAHI sample, corresponding to an incidence of 33.33%.

The meta-analysis by Adugna et al. [109] in broilers confirmed the negative effects of DON on performance, significantly reducing average daily feed intake and average daily weight gain, worsening fattening parameters such as feed conversion ratio, impairing small intestine health, including the structure of the duodenum, jejunum, and ileum, and negatively affecting immunoglobulin levels and antioxidant activity. Similarly, Kaur et al. [110] confirmed that maize silages are most heavily contaminated with deoxynivalenol, with an incidence of 100%. More recent data have also confirmed that DON is among the most frequently occurring mycotoxins in maize silage [111]. In the evaluated maize silages, the mean DON concentration ranged from 749.40 (BAHI) to 1312.20 (BALA) µg/kg, and in no treatment was the recommended value of 10,000 µg/kg established for maize and maize products exceeded, nor was the recommended value of 5000 µg/kg for complete and complementary feedstuffs for poultry exceeded, both at 12% moisture content [112]. Guan et al. [24] reported the positive effect of *L. plantarum* on the reduction in DON in fermented feeds by up to 90.61%. Li et al. [23] likewise reported that the addition of LAB positively affected the concentration of DON in maize silage. Specifically, *L. plantarum* was associated with lower DON concentration by 61.5%, *L. buchneri* by 64.8%, and the combination of *L. plantarum* + *L. buchneri* by 74.1% compared with the control.

The results of Wang et al. [113] in ducks confirmed the negative effect of fumonisin B1 on final live body weight and oviduct length, together with changes in serum biochemical indicators, oxidative stress, inflammatory, immune, and hormonal parameters, as well as histopathological lesions in the liver, kidneys, ovaries, and oviduct. Weaver et al. [114] showed that fumonisins belong among the prevalent *Fusarium* mycotoxins in corn silage and frequently co-occur with other mycotoxins. In a more recent seven-year European survey, Weaver et al. [111] likewise identified maize silage as a highly multi-contaminated feed and reported that fumonisin contamination varied according to region and harvest year. Similarly, Lapris et al. [115] confirmed the presence of fumonisins in corn silage from northern Italy. In the evaluated silage samples, FB1 values ranged from 155.09 (BATM) to 236.05 (BA) µg/kg and FB2 values from 113.98 (BATM) to 135.83 (BA) µg/kg, while the sum of fumonisins (FB1 + FB2) in none of the treatments exceeded the recommended value of 60,000 µg/kg established for maize and maize products at 12% moisture content, nor the guidance value of 20,000 µg/kg for complete and complementary feedstuffs for poultry [112]. A reduction in FB1 concentration by 66.5% after the application of *L. plantarum* in maize silage was reported by Li et al. [23], whereas Gallo et al. [22] reported 32.7% lower FB2 concentration after the application of a combination of *L. buchneri* and *L. lactis*.

The results of Gu et al. [116] in Yangzhou goslings confirmed the negative effect of T-2 toxin on growth performance, feather quality, tibial development, and blood indicators, together with signs of liver and kidney damage. In ducks, An et al. [117,118] showed that T-2 toxin induced hepatotoxicity, oxidative stress, disturbances in lipid metabolism, impairment of the intestinal barrier, and dysbiosis accompanied by subsequent hepatic inflammation. Zhang et al. [119] and, more recently, Weaver et al. [111] confirmed the presence of T-2 toxin in maize silage, although only at low prevalence and concentrations, with detection rates of 1.5% and 3.35% of samples, respectively. In both studies, HT-2 occurred more frequently and at higher levels than T-2. In this study, the presence of T-2 toxin was confirmed only in the BAHI sample, with an incidence of 33.33%. The observed T-2 toxin content in BAHI was 83.93 µg/kg. Current EU legislation does not establish a specific guidance value for the sum of T-2 + HT-2 specifically for maize silages or feed for poultry. For compound feed, with the exception of feed for cats, a non-binding indicative value of 250 µg/kg is applied [120].

Lauric acid has recognized antimicrobial activity, particularly against Gram-positive bacteria, although inhibitory effects against *E. coli* have also been reported [121]. Evidence regarding its antifungal activity is likewise available, including documented bioactivity against *Fusarium* spp. [122] and *Aspergillus flavus* [123]. In addition, the hydrolysis of urea releases ammonia, which exerts fungicidal effects. However, in maize silage, a clear antifungal effect of urea has not been consistently confirmed, as microscopic filamentous fungi remained detectable after urea application [106].

Despite the documented antimicrobial and antifungal activity of lauric acid, the treatments with lauric acid (BALA, BAHI) did not improve the microbiological counts of the maize silages, as no significant differences were observed in TVC, CB, MFF, or LAB compared with the other treatments. Likewise, BALA did not show a favourable effect on mycotoxin contamination as DON concentration remained among the highest, and no clear reduction was observed for FB1 or FB2. However, this interpretation should be made with caution, since lauric acid was also present in the BAHI treatment through *H. illucens*, and this treatment showed one of the lowest DON concentrations. Therefore, any potential effect of lauric acid in BAHI cannot be separated from the broader nutritional and compositional effects of *H. illucens*. Moreover, DON, FB1, and FB2 were not significantly correlated with MFF (Table 9), suggesting that the counts of MFF did not directly reflect the mycotoxin burden of the silages. In contrast, treatments with higher CP (BAUR, BAHI, BATM) content tended to show lower DON concentrations, and this pattern was strongly supported by the negative correlation between DON and CP (Table 9), suggesting that protein-enriched treatments were associated with lower DON content rather than providing direct evidence of causality. The results should also be interpreted with caution because the number of independent silage replicates per treatment was limited, which reduces the robustness of the observed treatment differences. Further research is therefore needed to clarify the mechanisms underlying the relationships between nitrogen enrichment, lauric acid supplementation in combination with LAB, and DON concentration in maize silage.

## 5. Conclusions

In this study, insect meal supplementation in combination with LAB improved selected compositional indicators of nutritional value of maize silage more consistently than urea or lauric acid in combination with LAB. Both insect variants, BAHI and BATM, increased CP compared with BA, whereas the increase in EE was observed only in the insect + LAB treated silage and was most pronounced in BAHI. Among all treatments, BAHI also showed the highest calculated AMEN, indicating the most favourable energy value. In addition, both insect + LAB variants improved the AA profile and protein quality, as reflected by higher EAAI values, with the strongest effect recorded in BATM, while BAUR increased CP without improving AA quality. None of the tested additives significantly affected the microbiological counts of the silages. In contrast, treatment-related differences were observed in the mycotoxin profile: lower DON concentrations were observed in BAUR, BAHI, and BATM, whereas lower FB1 concentrations were observed in BATM and BALA; FB2 was not significantly affected. Only DON, FB1, and FB2 were consistently detected across treatments, while T-2 was detected only in BAHI (incidence 33.33%). Aflatoxins, OTA, ZEA, and HT-2 were not detected.

These results suggest that the inclusion of small amounts of insect material in combination with LAB during maize ensiling improved selected compositional indicators of nutritional value of maize silage without adverse effects on the culture-based microbiological counts, and with selective effects on the mycotoxin profile. However, these findings should be interpreted within the limits of a compositional study, and the practical feeding value and the effects of higher inclusion levels still need to be confirmed in future in vivo studies.

## Figures and Tables

**Table 2:** Nutritional composition of the tested maize silages.

	BA	BAUR	BAHI	BATM	BALA
	g/kg				
DM	336.03 ^b^ ±2.81	334.33 ^b^ ±0.10	349.15 ^a^ ±0.17	350.28 ^a^ ±5.46	326.15 ^c^ ±2.66
	g/kg DM				
CP	49.73 ^b^ ±1.14	66.37 ^a^ ±1.82	64.94 ^a^ ±1.00	67.32 ^a^ ±0.58	47.81 ^b^ ±0.36
EE	21.74 ^c^ ±0.32	19.77 ^d^ ±0.46	40.08 ^a^ ±0.56	34.36 ^b^ ±0.99	20.87 ^cd^ ±0.70
NFE	719.72 ^a^ ±4.79	710.82 ^ab^ ±5.56	699.94 ^bc^ ±3.69	693.12 ^c^ ±2.45	707.52 ^ab^ ±11.73
NSCs	543.58 ^a^ ±4.66	531.71 ^a^ ±4.94	538.54 ^a^ ±1.35	497.73 ^b^ ±9.89	504.40 ^b^ ±16.86
Starch	275.05 ^a^ ±4.56	256.31 ^b^ ±0.71	242.79 ^cd^ ±4.65	240.98 ^d^ ±4.55	252.11 ^bc^ ±7.98
Ash	39.03 ^b^ ±0.40	38.85 ^b^ ±0.68	40.97 ^a^ ±0.28	37.35 ^c^ ±0.23	38.93 ^b^ ±0.73
OM	960.97 ^b^ ±0.40	961.15 ^b^ ±0.68	959.03 ^c^ ±0.28	962.65 ^a^ ±0.23	961.07 ^b^ ±0.73
	MJ/kg DM				
AMEN	9.14 ^b^ ±0.11	9.22 ^b^ ±0.07	9.87 ^a^ ±0.07	9.43 ^b^ ±0.11	8.55 ^c^ ±0.32

Abbreviations: BA—biological additive, BAUR—BA + urea, BAHI—BA + *H. illucens*, BATM—BA + *T. molitor* meal, BALA—BA + lauric acid, DM—dry matter, CP—crude protein, EE—ether extract, NFE—nitrogen free extract, NSCs—non-structural carbohydrates, OM—organic matter, AMEN—calculated nitrogen-corrected apparent metabolizable energy for poultry. Statistically significant differences were observed with different indexes (^abcd^) in rows (*p* < 0.05).

**Table 3:** Structural carbohydrate content of the tested maize silages.

	BA	BAUR	BAHI	BATM	BALA
	g/kg DM				
CF	169.79 ^b^ ±3.43	164.19 ^bc^ ±4.43	154.08 ^c^ ±2.87	167.86 ^b^ ±1.93	184.88 ^a^ ±9.96
NDF	345.93 ^bc^ ±3.86	343.30 ^c^ ±6.59	315.48 ^d^ ±1.59	363.25 ^b^ ±11.21	388.01 ^a^ ±15.11
ADF	184.89 ^bc^ ±5.92	190.31 ^b^ ±3.19	177.33 ^c^ ±6.86	185.11 ^bc^ ±4.21	206.30 ^a^ ±4.17
ADL	16.84 ^a^ ±1.09	12.50 ^b^ ±0.99	11.92 ^b^ ±0.94	16.71 ^a^ ±2.86	15.57 ^ab^ ±2.62
CEL	168.04 ^b^ ±6.59	177.81 ^b^ ±3.92	165.41 ^c^ ±7.44	168.40 ^b^ ±2.81	190.73 ^a^ ±3.54
HEM	161.04 ^ab^ ±3.88	152.99 ^bc^ ±5.73	138.15 ^c^ ±7.50	178.14 ^a^ ±15.00	181.71 ^a^ ±11.95

Abbreviations: BA—biological additive, BAUR—BA + urea, BAHI—BA + *H. illucens*, BATM—BA + *T. molitor* meal, BALA—BA + lauric acid, DM—dry matter, CF—crude fibre, NDF—neutral detergent fibre, ADF—acid detergent fibre, ADL—acid detergent lignin, CEL—cellulose, HEM—hemicellulose. Statistically significant differences were observed with different indexes (^abcd^) in rows (*p <* 0.05).

**Table 4:** Essential amino acid composition of the tested maize silages.

	BA	BAUR	BAHI	BATM	BALA
	g/kg DM				
Arg	1.18 ^c^ ±0.02	1.09 ^d^ ±0.01	1.63 ^b^ ±0.03	1.74 ^a^ ±0.03	1.04 ^d^ ±0.01
His	0.97 ^e^ ±0.00	1.15 ^d^ ±0.01	1.66 ^b^ ±0.02	1.80 ^a^ ±0.02	1.29 ^c^ ±0.01
Ile	1.42 ^c^ ±0.02	1.44 ^c^ ±0.03	2.18 ^a^ ±0.00	2.12 ^b^ ±0.00	1.28 ^d^ ±0.02
Leu	4.20 ^c^ ±0.03	4.07 ^d^ ±0.04	5.37 ^b^ ±0.02	5.71 ^a^ ±0.02	3.61 ^e^ ±0.01
Lys	1.62 ^e^ ±0.03	1.85 ^d^ ±0.01	3.09 ^a^ ±0.01	2.59 ^b^ ±0.01	1.92 ^c^ ±0.01
Met	1.41 ^c^ ±0.03	1.44 ^c^ ±0.01	3.28 ^b^ ±0.01	3.76 ^a^ ±0.02	1.41 ^c^ ±0.01
Phe	1.65 ^d^ ±0.02	1.74 ^c^ ±0.02	1.87 ^b^ ±0.03	2.94 ^a^ ±0.05	1.73 ^cd^ ±0.02
Thr	1.25 ^d^ ±0.02	1.54 ^c^ ±0.01	2.55 ^a^ ±0.02	1.59 ^b^ ±0.01	1.23 ^d^ ±0.01
Val	2.29 ^b^ ±0.04	2.04 ^d^ ±0.01	1.78 ^e^ ±0.02	3.26 ^a^ ±0.04	2.18 ^c^ ±0.02
SUMEAAs	15.99 ^cd^ ±0.20	16.35 ^c^ ±0.15	23.42 ^b^ ±0.16	25.51 ^a^ ±0.19	15.69 ^d^ ±0.12
	%				
EAAI_D_	60.65 ^c^ ±1.73	48.31 ^d^ ±0.50	69.52 ^b^ ±1.42	72.86 ^a^ ±0.98	63.75 ^c^ ±0.91
EAAI_G_	57.93 ^c^ ±1.65	46.15 ^d^ ±0.48	66.41 ^b^ ±1.35	69.60 ^a^ ±0.94	60.90 ^c^ ±0.87

Abbreviations: BA—biological additive, BAUR—BA + urea, BAHI—BA + *H. illucens*, BATM—BA + *T. molitor* meal, BALA—BA + lauric acid, DM—dry matter, Arg—arginine, His—histidine, Ile—isoleucine, Leu—leucine, Lys—lysine, Met—methionine, Phe—phenylalanine, Thr—threonine, Val—valine, SUMEAAs—total essential amino acids without tryptophan, EAAI_D_—essential amino acid index relative to duck egg protein, EAAI_G_—essential amino acid index relative to goose egg protein. Statistically significant differences were observed with different indexes (^abcde^) in rows (*p* < 0.05).

**Table 5:** Non-essential amino acid composition of the tested maize silages.

	BA	BAUR	BAHI	BATM	BALA
	g/kg DM				
Ala	3.85 ^c^ ±0.05	3.69 ^c^ ±0.07	4.92 ^b^ ±0.06	5.38 ^a^ ±0.06	3.82 ^c^ ±0.08
Asp	2.97 ^cd^ ±0.00	2.86 ^d^ ±0.04	4.20 ^b^ ±0.07	4.65 ^a^ ±0.07	3.04 ^c^ ±0.02
Cys	0.08 ^b^ ±0.00	0.09 ^a^ ±0.00	0.08 ^b^ ±0.00	0.08 ^b^ ±0.00	0.07 ^c^ ±0.00
Glu	6.64 ^c^ ±0.03	6.38 ^d^ ±0.03	8.77 ^b^ ±0.01	8.90 ^a^ ±0.01	6.25 ^e^ ±0.02
Gly	2.07 ^c^ ±0.01	1.96 ^d^ ±0.02	2.92 ^b^ ±0.03	3.13 ^a^ ±0.03	2.06 ^c^ ±0.01
Pro	5.62 ^b^ ±0.02	5.57 ^bc^ ±0.07	3.80 ^d^ ±0.01	5.42 ^c^ ±0.10	7.42 ^a^ ±0.08
Ser	1.21 ^e^ ±0.02	2.03 ^b^ ±0.03	2.64 ^a^ ±0.03	1.59 ^c^ ±0.02	1.43 ^d^ ±0.02
Tyr	0.76 ^cd^ ±0.00	0.81 ^c^ ±0.00	0.98 ^b^ ±0.02	2.20 ^a^ ±0.04	0.74 ^d^ ±0.01
SUMNEAAs	23.19 ^c^ ±0.14	23.39 ^c^ ±0.26	24.89 ^b^ ±0.22	31.35 ^a^ ±0.34	24.82 ^b^ ±0.25

Abbreviations: BA—biological additive, BAUR—BA + urea, BAHI—BA + *H. illucens*, BATM—BA + *T. molitor* meal, BALA—BA + lauric acid, DM—dry matter, Ala—alanine, Asp—aspartic acid, Cys—cysteine, Glu—glutamic acid, Gly—glycine, Pro—proline, Ser—serine, Tyr—tyrosine, SUMNEAAs—total non-essential amino acids. Statistically significant differences were observed with different indexes (^abcde^) in rows (*p* < 0.05).

**Table 6:** Fatty acid composition of the tested maize silages.

	BA	BAUR	BAHI	BATM	BALA
	% of total FA				
Capric acid C10:0	N.D.	N.D.	0.46 ±0.01	N.D.	N.D.
Lauric acid C12:0	0.32 ^c^ ±0.02	0.43 ^c^ ±0.02	11.86 ^a^ ±0.34	0.30 ^c^ ±0.00	1.81 ^b^ ±0.12
Myristic acid C14:0	N.D.	N.D.	2.41 ^a^ ±0.05	1.26 ^b^ ±0.01	N.D.
Palmitic acid C16:0	12.10 ^d^ ±0.09	12.36 ^c^ ±0.06	15.10 ^a^ ±0.06	14.48 ^b^ ±0.03	11.95 ^e^ ±0.04
Palmitoleic acid C16:1	N.D.	N.D.	1.00 ^a^ ±0.02	0.77 ^b^ ±0.01	N.D.
Stearic acid C18:0	2.45 ^dc^ ±0.04	2.50 ^c^ ±0.03	2.97 ^a^ ±0.04	2.66 ^b^ ±0.02	2.39 ^d^ ±0.01
Oleic acid C18:1	25.82 ^b^ ±0.22	25.94 ^b^ ±0.26	24.95 ^c^ ±0.20	31.82 ^a^ ±0.14	24.67 ^c^ ±0.20
Linoleic acid C18:2 n-6	49.35 ^a^ ±0.18	49.24 ^a^ ±0.12	33.00 ^d^ ±0.32	40.93 ^c^ ±0.29	48.33 ^b^ ±0.09
α-linoleic acid C18:3 n-3	6.77 ^b^ ±0.17	6.63 ^b^ ±0.07	4.16 ^d^ ±0.05	4.88 ^c^ ±0.21	7.44 ^a^ ±0.06
Arachidic acid C20:0	0.63 ^ab^ ±0.02	0.65 ^a^ ±0.01	0.33 ^d^ ±0.01	0.40 ^c^ ±0.01	0.61 ^b^ ±0.02
cis-11-eicosenoic acid C20:1	N.D.	N.D.	0.22 ±0.00	N.D.	N.D.
Behenic acid C22:0	0.40 ^a^ ±0.02	0.44 ^a^ ±0.02	0.20 ^b^ ±0.01	N.D.	0.41 ^a^ ±0.02
SUMunidentified	2.16 ^c^ ±0.03	1.82 ^d^ ±0.04	3.34 ^a^ ±0.06	2.51 ^b^ ±0.15	2.40 ^b^ ±0.04
UFAs	81.94 ^a^ ±0.11	81.81 ^a^ ±0.11	63.33 ^d^ ±0.36	78.39 ^c^ ±0.18	80.44 ^b^ ±0.19
MUFAs	25.82 ^b^ ±0.22	25.94 ^b^ ±0.26	26.17 ^b^ ±0.20	32.58 ^a^ ±0.14	24.67 ^c^ ±0.20
PUFAs	56.12 ^a^ ±0.19	55.87 ^a^ ±0.18	37.15 ^c^ ±0.37	45.81 ^b^ ±0.17	55.77 ^a^ ±0.08
SFAs	15.90 ^e^ ±0.08	16.37 ^d^ ±0.07	33.33 ^a^ ±0.31	19.10 ^b^ ±0.04	17.16 ^c^ ±0.16
n3/n6	0.14 ^b^ ±0.00	0.13 ^b^ ±0.00	0.13 ^c^ ±0.00	0.12 ^c^ ±0.01	0.15 ^a^ ±0.00
n6/n3	7.29 ^c^ ±0.21	7.42 ^c^ ±0.06	7.94 ^b^ ±0.04	8.40 ^a^ ±0.41	6.49 ^d^ ±0.06

Abbreviations: BA—biological additive, BAUR—BA + urea, BAHI—BA + *H. illucens*, BATM—BA + *T. molitor* meal, BALA—BA + lauric acid, UFAs—unsaturated fatty acids, MUFAs—monounsaturated fatty acids, PUFAs—polyunsaturated fatty acids, SFAs—saturated fatty acids, N.D.—not detected. Statistically significant differences were observed with different indexes (^abcde^) in rows (*p* < 0.05).

**Table 7:** Selected microbiological indicators of the tested maize silages.

	BA	BAUR	BAHI	BATM	BALA
	log CFUs/g				
TVC	1.52 ±0.06	1.42 ±0.07	1.53 ±0.20	1.43 ±0.12	1.89 ±0.70
LAB	1.38 ±0.10	1.36 ±0.12	1.50 ±0.14	1.45 ±0.11	1.36 ±0.09
CB	1.33 ±0.10	1.41 ±0.06	1.36 ±0.02	1.33 ±0.10	1.30 ±0.06
MFF	1.40 ±0.06	1.62 ±0.16	1.51 ±0.22	1.44 ±0.10	1.59 ±0.27

Abbreviations: BA—biological additive, BAUR—BA + urea, BAHI—BA + *H. illucens*, BATM—BA + *T. molitor* meal, BALA—BA + lauric acid, CFU—colony forming units, DM—dry matter, TVC—total viable count, CB—coliform bacteria, LAB—lactic acid bacteria, MFF—microscopic filamentous fungi. No statistically significant differences were observed among treatments (*p* > 0.05).

**Table 8:** Mycotoxin content and incidence in the tested maize silages.

	BA	BAUR	BAHI	BATM	BALA
	µg/kg 88% DM				
FB1	236.05 ^a^ ±15.52 (100) *	225.31 ^a^ ±20.44 (100) *	199.20 ^ab^ ±12.25 (100) *	155.09 ^c^ ±2.91 (100) *	182.03 ^bc^ ±20.65 (100) *
FB2	135.83 ±10.91 (100) *	126.70 ±3.48 (100) *	126.52 ±13.12 (100) *	113.98 ±5.16 (100) *	119.44 ±4.32 (100) *
ΣAFs	N.D.	N.D.	N.D.	N.D.	N.D.
OTA	N.D.	N.D.	N.D.	N.D.	N.D.
ZEA	N.D.	N.D.	N.D.	N.D.	N.D.
T-2	N.D.	N.D.	83.93 (33.33) *	N.D.	N.D.
HT-2	N.D.	N.D.	N.D.	N.D.	N.D.
DON	1198.56 ^a^ ±23.76 (100) *	754.56 ^c^ ±56.72 (100) *	749.40 ^c^ ±30.28 (100) *	874.28 ^b^ ±54.75 (100) *	1312.20 ^a^ ±43.87 (100) *

Abbreviations: BA—biological additive, BAUR—BA + urea, BAHI—BA + *H. illucens*, BATM—BA + *T. molitor* meal, BALA—BA + lauric acid, DM—dry matter, FB1—fumonisin B1, FB2—fumonisin B2, ΣAFs—total aflatoxins, OTA—ochratoxin A, ZEA—zearalenone, T-2—T2-toxin, HT-2—HT-2 toxin, DON—deoxynivalenol, N.D.—not detected. * Values in parentheses indicate mycotoxin incidence (% of positive samples), 100 = detection in all tested samples of the respective variant (3/3). Statistically significant differences were observed with different indexes (^abc^) in rows (*p* < 0.05).

**Table 9:** Selected correlations supporting the interpretation of treatment effects in the tested maize silages.

Variable 1	Variable 2	*r*	*p*
DON	CP	−0.941	<0.001
CP	SUMEAAs	0.725	0.001
DON	NDF	0.713	0.001
ADF	EE	−0.614	0.002
DON	HEM	0.654	0.004
DON	ADF	0.607	0.008
FB1	FB2	0.598	0.009
NDF	EE	−0.500	0.012
NDF	CP	−0.495	0.013
DON	MFF	−0.156	0.289
FB1	MFF	0.041	0.443
FB2	MFF	−0.331	0.114

Abbreviations: DON—deoxynivalenol, FB1—fumonisin B1, FB2—fumonisin B2, CP—crude protein, EE—ether extract, ADF—acid detergent fibre, NDF—neutral detergent fibre, HEM—hemicellulose, SUMEAAs—sum of essential amino acids.

## Data Availability

The data presented in this study are available from the corresponding author on request.
